# Protein arginine methyltransferase 1 regulates mouse enteroendocrine cell development and homeostasis

**DOI:** 10.1186/s13578-024-01257-x

**Published:** 2024-06-04

**Authors:** Zhaoyi Peng, Lingyu Bao, James Iben, Shouhong Wang, Bingyin Shi, Yun-Bo Shi

**Affiliations:** 1 Department of Endocrinology, The First Affiliated Hospital of Xi’an JiaoTong University, No. 277, West Yanta Road, Xi’an, 710061 Shaanxi China; 2 Section on Molecular Morphogenesis, Eunice Kennedy Shriver National Institute of Child Health and Human Development, National Institutes of Health, Bethesda, MD 20892 USA; 3 Molecular Genomics Core, Eunice Kennedy Shriver National Institute of Child Health and Human Development, National Institutes of Health, Bethesda, MD 20892 USA; 4 Chengdu Institute of Biology, Chinese Academy of Sciences, Chengdu, 610041 China

**Keywords:** Enteroendocrine cell, Methyltransferase, RNA-Seq, Transcription, Neurogenin 3, Differentiation

## Abstract

**Background:**

The adult intestinal epithelium is a complex, self-renewing tissue composed of specialized cell types with diverse functions. Intestinal stem cells (ISCs) located at the bottom of crypts, where they divide to either self-renew, or move to the transit amplifying zone to divide and differentiate into absorptive and secretory cells as they move along the crypt-villus axis. Enteroendocrine cells (EECs), one type of secretory cells, are the most abundant hormone-producing cells in mammals and involved in the control of energy homeostasis. However, regulation of EEC development and homeostasis is still unclear or controversial. We have previously shown that protein arginine methyltransferase (PRMT) 1, a histone methyltransferase and transcription co-activator, is important for adult intestinal epithelial homeostasis.

**Results:**

To investigate how PRMT1 affects adult intestinal epithelial homeostasis, we performed RNA-Seq on small intestinal crypts of tamoxifen-induced intestinal epithelium-specific PRMT1 knockout and PRMT1^fl/fl^ adult mice. We found that PRMT1^fl/fl^ and PRMT1-deficient small intestinal crypts exhibited markedly different mRNA profiles. Surprisingly, GO terms and KEGG pathway analyses showed that the topmost significantly enriched pathways among the genes upregulated in PRMT1 knockout crypts were associated with EECs. In particular, genes encoding enteroendocrine-specific hormones and transcription factors were upregulated in PRMT1-deficient small intestine. Moreover, a marked increase in the number of EECs was found in the PRMT1 knockout small intestine. Concomitantly, Neurogenin 3-positive enteroendocrine progenitor cells was also increased in the small intestinal crypts of the knockout mice, accompanied by the upregulation of the expression levels of downstream targets of Neurogenin 3, including Neuod1, Pax4, Insm1, in PRMT1-deficient crypts.

**Conclusions:**

Our finding for the first time revealed that the epigenetic enzyme PRMT1 controls mouse enteroendocrine cell development, most likely via inhibition of Neurogenin 3-mediated commitment to EEC lineage. It further suggests a potential role of PRMT1 as a critical transcriptional cofactor in EECs specification and homeostasis to affect metabolism and metabolic diseases.

**Supplementary Information:**

The online version contains supplementary material available at 10.1186/s13578-024-01257-x.

## Background

The adult intestinal epithelium is a complex, self-renewing tissue composed of specialized cell types with diverse functions. Intestinal stem cells (ISCs) located at the bottom of crypts, divide to either self-renew, or move to the transit amplifying zone to divide and differentiate into absorptive and secretory cells [1–5]. Enteroendocrine cells (EECs), one type of secretory cells, are the most abundant hormone-producing cells in mammals and constitute the largest endocrine system in the body. EECs secrete various hormones including glucagon-like peptides1 and 2 (GLP-1, GLP-2), peptide YY (PYY), cholecystokinin (CCK), gastric inhibitory polypeptide (GIP), secretin (SCT), Ghrelin (GHRL), neurotensin (NTS) and neurotransmitter serotonin (5-HT). These EEC-derived gut hormones and peptides play crucial roles in regulating physiological functions such as nutrient intake, lipid adsorption, glucose homeostasis, and gut motility [6–10]. EECs are often dysregulated in obesity and type 2 diabetes patients [11, 12]. Importantly, GLP-1 receptor agonists are already widely used to treat diabetes and obesity, and emerging gut hormone-based combination therapies provide a new option for metabolic diseases [13–15]. As more therapeutics are being designed to target EEC hormones, a comprehensive understanding of the differentiation and function of the EEC system becomes essential.

Like all mature intestinal epithelial cells, mouse EECs are actively renewed every 3–5 days throughout adult life. They originate from ISCs and transit-amplifying cells, the highly proliferative progenitor cells derived from ISCs [1–3], and commit into secretory cell lineage under the control of the transcription factor mouth atonal homolog 1 (Atoh1) [16]. Subsequently, transcription factor Neurogenin 3 (Neurog3) promotes enteroendocrine lineage differentiation. Neurog3 is transiently expressed in early secretory progenitors, and its expression is turned off as the cells become post-mitotic and differentiate into EECs [17–19]. Neurog3 initiates the differentiation of subpopulations of epithelial cells by activating a cascade of downstream target genes including Neurod1 [20], Pax4/6 [21, 22], Arx [21], and Insm1 [23]. Mice lacking Neurog3 in the intestinal epithelium do not have any EECs and gut hormones, with growth retardation and increased lethality [24]. Similarly, humans with mutations in Neurog3 have impaired EEC development after birth and have severe malabsorptive diarrhea [25]. Neurog3 dosage regulates the allocation of intestinal cell fate toward EEC vs goblet cells, the mucus-producing secretory cells [26], indicating a reciprocal interaction in the determination of secretory cell fate. Recently, novel transcriptional regulators of enteroendocrine differentiation have been identified by single-cell RNA and/or ATAC seq in both mouse [27, 28] and human [29, 30]. However, the upstream regulators of Neurog3 and how those downstream transcription factors coordinately interact with each other to generate mature EEC types remain unknown.

Protein arginine methyltransferase 1 (PRMT1) is the predominant arginine methyltransferase in mammalian cells and responsible for over 85% of arginine methylation activity [31]. Substrates of PRMT1 include histones H3 and H4, transcription factors, and other cellular signaling proteins [32–34]. PRMT1 has been reported to play critical roles in various physiological processes as a result of its diverse substrates [35]. For example, it has been demonstrated that PRMT1 is involved in regulating normal development of lymphocytes (B cells) [36] and identity of mature β-cell [37]. We have previously found that intestinal epithelium-specific PRMT1 knockout leads to mouse intestinal defects in the adult due to dysregulation of intestinal homeostasis [38, 39]. In addition, PRMT1 is highly expressed in mouse crypt epithelium, where proliferating cells and ISCs are located [38–40].

Here, we attempted to investigate the potential molecular mechanism underlying PRMT1 function in adult intestine by using tamoxifen-inducible intestinal epithelium-specific PRMT1 knockout mice for RNA-Seq analysis. Our results showed PRMT1^fl/fl^ and PRMT1-knockout small intestinal crypts exhibited markedly different mRNA expression profiles. Surprisingly, GO and KEGG pathway analyses of the differentially expressed genes (DEGs) between PRMT1^fl/fl^ and knockout crypts showed that the topmost significantly enriched GO terms and biological pathways among the upregulated genes in PRMT1 knockout crypts were associated with EECs. Consistently, there was a marked increase in the number of EECs in PRMT1 knockout small intestine. We also observed that Neurog3-positive enteroendocrine progenitor cells increased in the small intestinal crypts of the knockout mice, accompanied by increased expression of downstream target genes of Neurog3. Our findings thus uncover a previously unknown role of the epigenetic enzyme PRMT1 in controlling mouse EEC development and homeostasis.

## Results

### Transcriptomic changes due to inducible knockout of PRMT1 in adult intestinal epithelium.

**Fig. 1:**
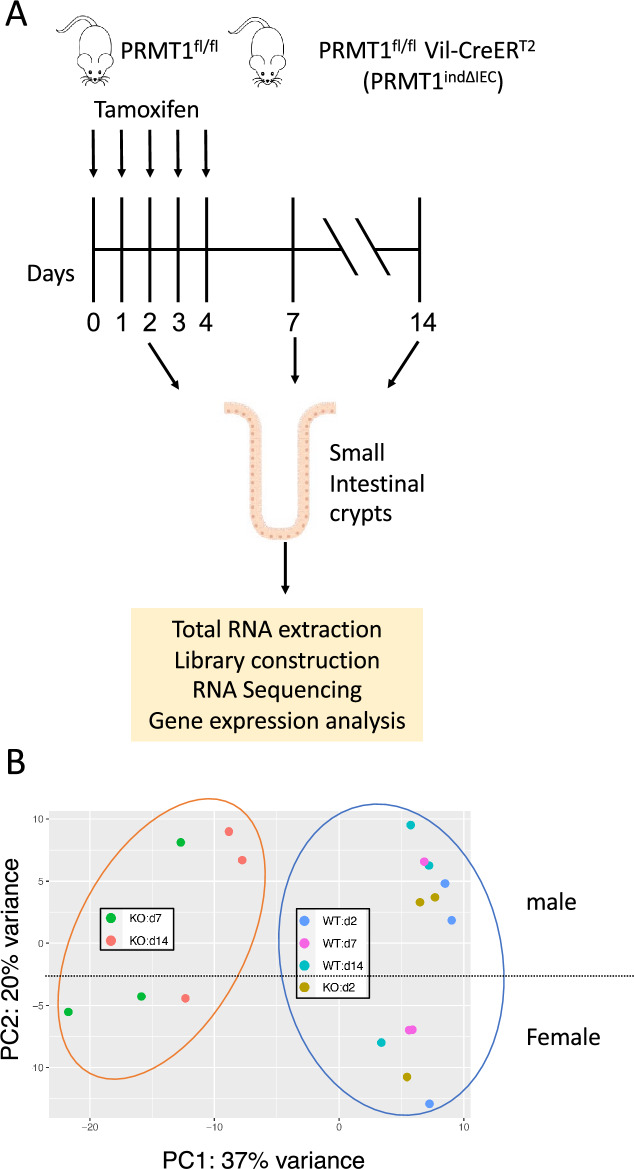
Induced deletion of PRMT1 from adult intestinal epithelium leads to robust transcriptomic changes. **A** Schematic diagram for inducible PRMT1 deletion in adult intestinal epithelium with tamoxifen treatment and time points at which RNA-Seq was performed. The PRMT1^fl/fl^ and PRMT1^indΔIEC^ mice were injected with 2 mg tamoxifen for 5 consecutive days (day 0–4). The intestine was collected at day 2 before the next tamoxifen injection, day 7 or day 14 as indicated. Small intestinal crypts were purified and used for RNA-Seq. Age- and gender- matched PRMT1^fl/fl^ littermates were used as control at each time point. n = 3 per group. **B** Significant changes in the transcriptome occurs at days 7 and 14 after initiating inducible PRMT1 knockout. Principal component analysis (PCA) of the RNA-Seq data from individual mice revealed that the 6 PRMT1^indΔIEC^ samples for days 7 and 14 (KO: d7 or d14) had very distinct spatial locations, well-separated from the rest of the samples along PC1 axis. Note that all tamoxifen-treated PRMT1^fl/fl^ (referred to as WT) and 2 day tamoxifen-treated PRMT1^indΔIEC^ (KO: d2) samples (n = 12) clustered together along PC1 axis. Interestingly, male samples (top; n = 10) and female samples (bottom; n = 8) were separated from each other along PC2 axis but not PC1 axis, indicating that there are distinct male and female gene expression patterns but PRMT1 KO has no gender-dependent effects

We previously generated a tamoxifen-induced intestinal epithelium-specific PRMT1 knockout mouse model (PRMT1^fl/fl^;Vil-CreER^T2^; henceforth referred to as PRMT1^indΔIEC^) and observed that induced PRMT1 deletion in mice of 8–12 weeks old led to abnormal intestine, including increased cell proliferation and longer crypts in the small intestine compared to PRMT1^fl/fl^ mice [38]. To provide insight into the mechanisms underlying the changes resulted from PRMT1 knockout, we performed RNA-Seq on small intestinal crypts from PRMT1^indΔIEC^ (KO) and PRMT1^fl/fl^ mice at different time points after the first tamoxifen injection (Fig. 1A). The RNA-Seq data from individual samples were assessed by principal component analysis (PCA). We observed a distinct separation of tamoxifen-treated day 7 and 14 KO samples from the rest of the samples, including day 2 KO samples, along PC1 axis on the PCA map (Fig. 1B). This suggests that 2 days tamoxifen-treated PRMT1^indΔIEC^ and PRMT1^fl/fl^ small intestinal crypts had similar transcriptome profiles and that different lengths of tamoxifen treatment did not affect the transcriptome profiles of PRMT1^fl/fl^ small intestinal crypts significantly. Consistently, there were few differentially expressed genes (DEGs) between 2 days tamoxifen-treated PRMT1^indΔIEC^ and PRMT1^fl/fl^ small intestinal crypts (Fig. S1A, Table S1). Interestingly, on the PCA map, we also observed a spatial separation between male (top) and female (bottom) samples among all 18 samples along PC2 axis (Fig. 1B). Consistently, we found a number of gender-specific DEGs (Table S2) and also observed that female and male samples had nearly identical transcriptome profiles except these gender-specific genes, as visualized by MA plot (Fig. S1B). This indicates there was no gender effect of PRMT1 knockout. Thus, we removed these gender-specific genes in the rest of the analyses.

**Fig. 2:**
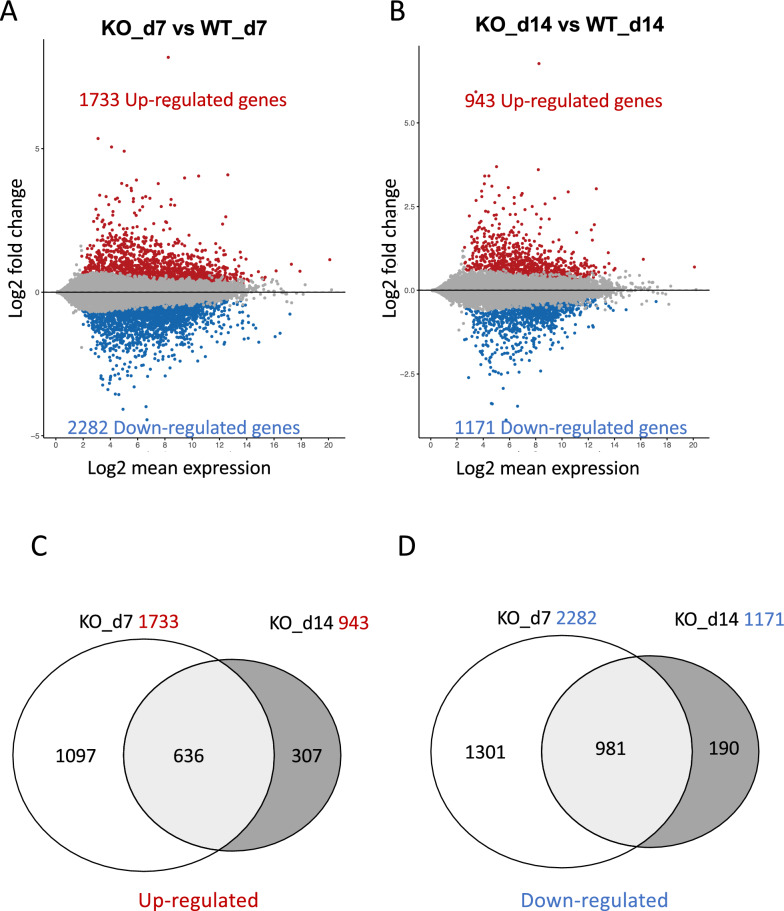
Differentially expressed genes (DEGs) after inducible PRMT1 KO in adult small intestinal crypts. **A**, **B** DEG analysis of the small intestinal crypts of adult PRMT1^fl/fl^ (WT) and PRMT1^indΔIEC^ (KO) mice at day 7 (**A**) and day 14 (**B**) after the initiation of tamoxifen treatment as in Fig. 1. MA plot visualizing the log2-fold change (M values) differences according to log2-mean expression levels (A values). Red and blue dots represent significantly (Adjusted p-value < 0.05) up- and down-regulated genes, respectively, in the KO compared to PRMT1^fl/fl^ intestine. Note that there were many more DEGs at day 7 compared to day 14, with 1733 up-regulated and 2282 down-regulated genes in at day 7 vs 943 up-regulated and 1171 down-regulated genes at day 14. **C**, **D** Venn diagrams depicting overlaps between DEGs after 7 and 14 days of tamoxifen treatment for the up- (**C**) or down- (**D**) regulated DEGs, respectively. Note that most of the upregulated (**C**) or downregulated (**D**) DEGs at day 14 were similarly regulated on day 7. WT: PRMT1^fl/fl^, KO: PRMT1^indΔIEC^

Next, we focused on day 7 and day14 samples as they exhibited distinct transcriptome profiles (Fig. 1B). We used DESeq2 for pairwise analyses to obtain DEGs between PRMT1^indΔIEC^ and PRMT1^fl/fl^ mice at day 7 or day 14 of tamoxifen treatment (Tables S3, S4). We identified 1733 up-regulated and 2282 down-regulated DEGs (p-adjusted < 0.05) at day 7 (Fig. 2A) and these numbers decreased to 943 up-regulated DEGs and 1171 down-regulated DEGs, respectively, at day 14 (Fig. 2B). This reduction suggests that some of transcriptome changes after tamoxifen treatment might be transient. To further explore this, we performed Venn diagram analyses on up-regulated (Fig. 2C) and down-regulated DEGs (Fig. 2D). We observed that the majority of the up-regulated (67.4%) or down-regulated (83.8%) DEGs on day 14 were also found on day 7, whereas most of the up- and down-regulated DEGs were exclusively on day 7 (Fig. 2C, D), suggesting that their regulation was transient. Interestingly, we noticed that stem cell markers such as lgr5 and olfm4 were among the transiently downregulated DEGs (Table S3). This observation might be related to the transient toxicity of tamoxifen to stems cells in the gastrointestinal tracts as reported before [41].

### PRMT1 deletion in adult intestinal epithelium affects pathways involved in enteroendocrine cells (EECs)

**Fig. 3:**
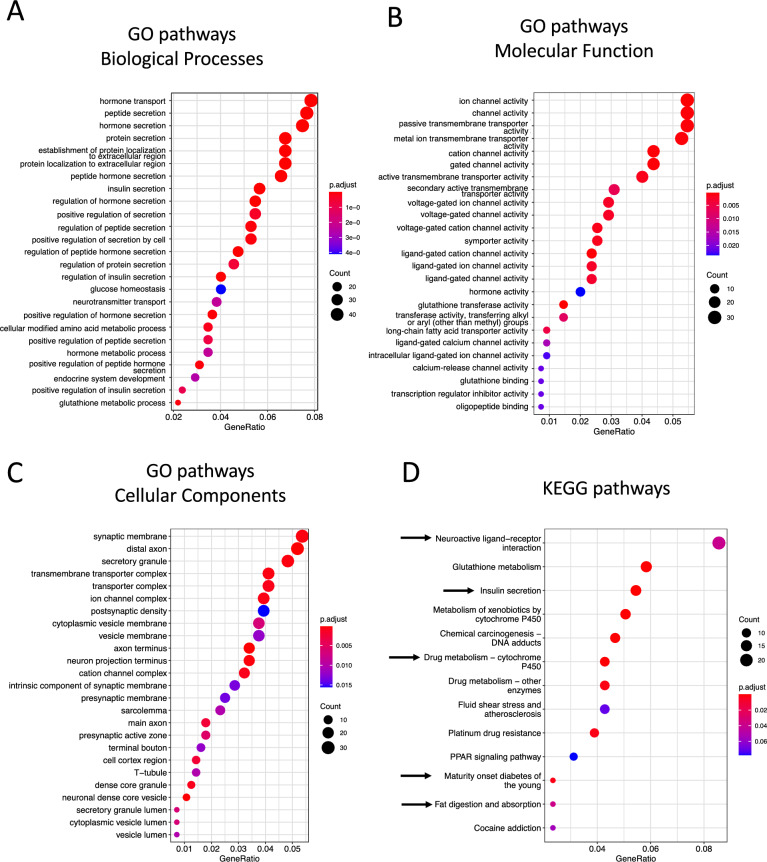
KEGG pathway and GO analyses reveal that PRMT1 deletion affects most significantly enteroendocrine cells (EECs). **A**–**C** The top 25 GO terms for biological processes (**A**), molecular function (**B**), and cellular components (**C**) that were significantly enriched among the up-regulated DEGs between PRMT1^indΔIEC^ (KO)_d14 and PRMT1^fl/fl^ (WT)_d14 intestinal crypts. All GO terms are involved in EECs. **D** The top 13 KEGG pathways significantly enriched among the up-regulated DEGs between PRMT1^indΔIEC^ (KO)_d14 and PRMT1^fl/fl^ (WT)_d14 intestinal crypts. Arrows point to pathways related to regulation of lipid and glucose metabolism. GO: Gene Ontology; KEGG: Kyoto Encyclopedia of Genes and Genomes. Horizontal axis: ratio of genes in a GO term or KEGG pathway to the totally number of up-regulated DEGs. From bottom to top along the vertically axis: increased gene counts in a GO term or KEGG pathway (size of the dot). The color of the dots indicates p-adj value for the enrichment

To reveal the effects of PRMT1 knockout and avoid potential transient effects on transcriptome induced by tamoxifen toxicity, we carried out pathway enrichment analyses by focusing on the up-regulated DEGs (p-adjusted < 0.05) on day 14 and obtained many GO terms and KEGG pathways enriched among the DEGs (Table S5–8). Surprisingly, the top enriched GO terms for biological function were associated with hormone secretion and regulation, glucose homeostasis and endocrine system development (Fig. 3A). In addition, the top enriched molecular function GO terms were primarily linked to ion channel activity, vesicle-mediated transporters activity, and hormone activity and metabolism (Fig. 3B). Finally, the top enriched cellular components GO terms were associated with vesicle membrane, secretory granule activity, and neuron projections (Fig. 3C). All these most significantly enriched GO terms are related to known properties and functions of the hormone-producing enteroendocrine cell (EEC), including synthesis, maturation, transport, storage, and secretion of hormones [7–10, 26]. Indeed, these pathways enriched among DEGs upregulated in PRMT1-deficient crypts coincide with pathways affected by Neurog3, a regulator for EEC development [26]. Similarly, KEGG pathway analysis showed that these up-regulated DEGs were enriched in neuroactive ligand-receptor interaction, insulin secretion, and fat digestion and absorption (Fig. 3D), again consistent with increased EEC function. Of note, the pathway for cytochrome P450, which has been shown to protect mice against HFD-induced obesity [42], was also significantly enriched among the DEGs, suggesting a potential role of PRMT1 in lipid metabolism. Taken together, these analyses unexpectedly revealed that PRMT1 deletion in adult intestinal epithelium affected pathways involved in EECs, suggesting PRMT1 may contribute to EEC development and function.

### The expression of enteroendocrine-specific hormone and transcription factor genes is upregulated in PRMT1^indΔIEC^ small intestinal epithelium

**Fig. 4:**
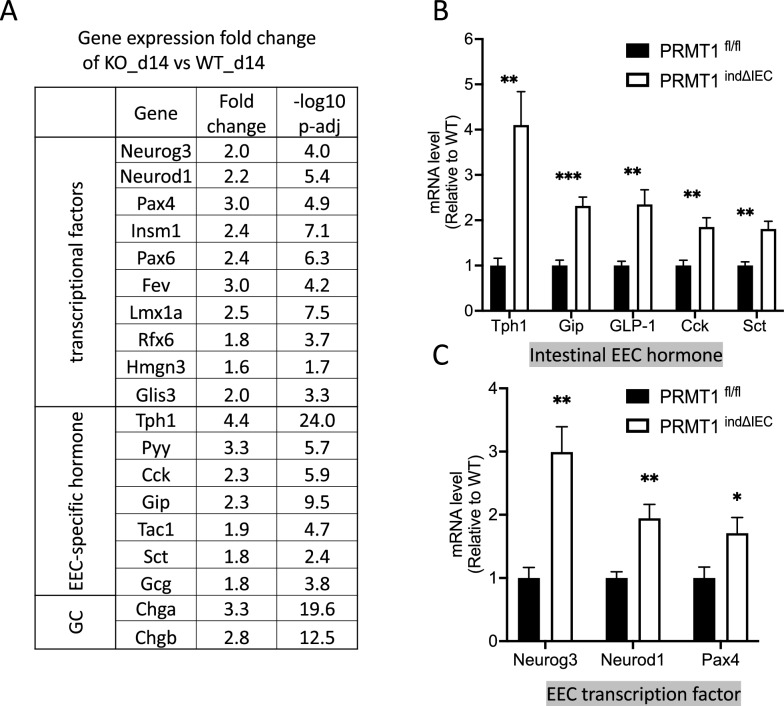
The expression of enteroendocrine-specific hormone and transcription factors are upregulated in PRMT1^indΔIEC^ small intestinal epithelium. **A** The expression of up-regulated enteroendocrine markers (transcriptional factors, EEC-specific hormones and granule components) were obtained from the RNA-seq data and the fold change between PRMT1^indΔIEC^ (KO)_d14 and PRMT1^fl/fl^ (WT)_d14 small intestinal crypts were presented with P-adj values in the table. **B**, **C** RT-PCR validation of the expression of intestinal EEC hormone genes (**B**) and EEC transcription factors (**C**) in the small intestinal epithelial cells of PRMT1^indΔIEC^ and PRMT1^fl/fl^ mice at day 14 after the first tamoxifen injection. The values were presented as mean ± SEM with n = 5 mice per group. *p < 0.05, **p < 0.01, ***p < 0.001. GC, granule components

To determine if EEC development and/or homeostasis is altered by PRMT1 deletion, we analyzed the expression levels of enteroendocrine markers. We found that indeed, the upregulated DEGs due to PRMT1 knockout included genes encoding intestinal peptide hormones or related key enzymes including Pyy, Cck, Gip, Sct, substance P (Tachykinin, Precursor 1(Tac1), Glp-1 precursor glucagon (Gcg) and tryptophan hydroxylase 1 (Tph1), a rate-limiting enzyme in serotonin biosynthesis, and granule components (Chga, Chgb) (Fig. 4A). In addition, the expression of transcription factors known to control EEC development and differentiation, including Neurog3, Neurod1, Pax4/6, Insm1, Fev and Lmx1a [43], were also significantly upregulated in PRMT1^indΔIEC^ small intestinal crypts (Fig. 4A). Consistent with recent studies reporting the identification of novel transcription factors, including Rfx6, Hmgn3, and Glis3, involved in EEC development [26, 44], we found that the expression of these three genes was upregulated in PRMT1 knockout crypts (Fig. 4A). To confirm the RNA-Seq findings, we analyzed the expression of several enteroendocrine markers and transcription factors independently by RT-qPCR. Consistently, the levels of the intestinal hormone genes, including Tph1, Gip, GLP-1, Cck and Sct (Fig. 4B) and transcription factor including Neurog3, Neurod1 and Pax4 (Fig. 4C), were significantly elevated in the small intestinal epithelium of PRMT1^indΔIEC^ mice compared to those in littermate controls at day 14 after the first tamoxifen injection. Therefore, our results suggest that PRMT1 controls transcriptional programs that repress the differentiation of enteroendocrine cells in the small intestine.

### The Number of enteroendocrine cells (EECs) is increased in the PRMT1-deficient adult small intestine

**Fig. 5:**
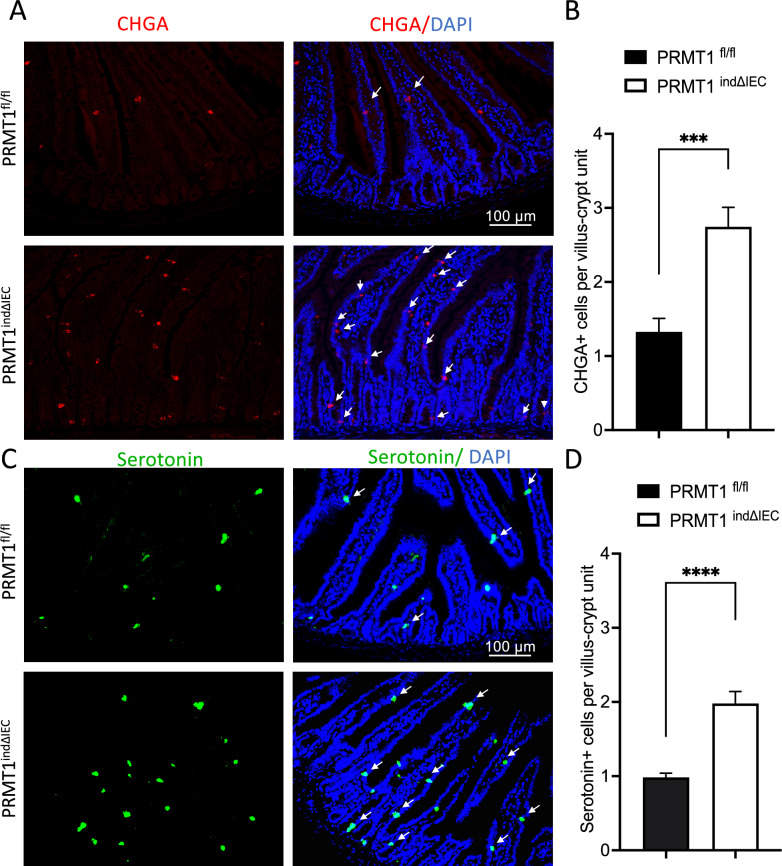
Inducible deletion of PRMT1 in the intestinal epithelium dramatically increases enteroendocrine cells (EECs) in adult small intestine. **A** Immunofluorescent staining for CHGA in small intestinal cross-sections from PRMT1^indΔIEC^ and PRMT1^fl/fl^ littermates at day 14 after the first tamoxifen injection as diagramed in Fig. 1A. The CHGA-labeling stained EEC cells red as indicated by arrows, and the DNA was stained blue with DAPI. **B** Quantification of CHGA + cells showed that PRMT1 deletion in adult intestinal epithelium dramatically increased the number of EECs. Multiple sections per animal were analyzed for each group. The values were presented as mean ± SEM with n = 3–4 mice per group. ***p < 0.001. Scale bars; 100 μm. CHGA: Chromogranin. **C** Immunofluorescent staining for serotonin in small intestinal cross-sections from PRMT1^indΔIEC^ and PRMT1^fl/fl^ littermates at day 14 after the first tamoxifen injection as diagramed in Fig. 1A. The serotonin-positive cells were stained green as indicated by arrows, and the DNA was stained blue with DAPI. **D** Quantification of serotonin-positive cells showed that PRMT1 deletion in adult intestinal epithelium increased the number of serotonin-positive cells, the most abundant subtype of EECs. Multiple sections per animal were analyzed for each group. The values were presented as mean ± SEM with n = 3–4 mice per group. ****p < 0.0001. Scale bars; 100 μm

The transcriptome and RT-PCR analyses above revealed a novel and unexpected role of PRMT1 in EEC development and/or function, further suggesting that the number of EECs might be affected by PRMT1 deletion. We analyzed EECs in small intestinal cross-sections by using immunofluorescent staining of chromogranin A (CHGA), an intestinal EEC marker. As shown in Fig. 5A, EECs were rare and primarily located in the villi in PRMT1^fl/fl^ mice. A dramatical increase in the number of EECs was observed in the small intestine of PRMT1^indΔIEC^ mice on day14 after the initiation of tamoxifen treatment, and the EECs were presented both in villi and crypts of PRMT1^indΔIEC^ small intestine (Fig. 5A). Quantitative analysis showed that the number of EECs in the small intestine of PRMT1^indΔIEC^ mice was about 2.5-fold of those in PRMT1^fl/fl^ mice (Fig. 5B). To further investigate the effect of PRMT knockout on EECs in the small intestine, we detected serotonin-positive enterochromaffin cells, the most abundant subtype of EECs, by using immunohistochemistry with an anti-serotonin antibody on small intestinal cross-sections (Fig. 5C). Consistently, the number of serotonin positive cells in the small intestine of PRMT1^indΔIEC^ mice was about twofold of those in PRMT1^fl/fl^ mice (Fig. 5D).

To further investigate the role of PRMT1 on EECs, we crossed PRMT1^fl/fl^ mice with Vil-cre mice, which expresses Cre recombinase under the control of intestinal epithelium specific villin promoter, to deleted PRMT1 in the intestinal epithelium during embryonic development and throughout adulthood (PRMT1^fl/fl^;Vil-Cre, henceforth referred to as PRMT1^ΔIEC^) [38, 39]. We found that the resulting adult PRMT1^ΔIEC^ had markedly increased CHGA+ cells in the small intestine compared to PRMT1^fl/fl^ mice (Fig. S2). We previously reported that intestinal epithelium-specific knockout of PRMT1 caused distinct, region-specific effects on the small intestine and colon: i.e., increasing and decreasing the goblet cell number in the small intestinal and colonic crypts, respectively [38]. To investigate whether PRMT1 knockout also has a region-specific effect on EECs, we detected EECs with anti-CHGA antibody on colonic sections of PRMT1^indΔIEC^ and PRMT1^fl/fl^ mice on day14 after the initiation of tamoxifen treatment (Fig. S3A). Interestingly, the number of EECs of PRMT1^indΔIEC^ colon was comparable to that of PRMT1^fl/fl^ colon (Fig. S3B). Furthermore, CHGA mRNA level in colonic epithelium was similar between PRMT1^indΔIEC^ and PRMT1^fl/fl^ mice (Fig. S3C). In addition, we observed that tamoxifen-induced knockout PRMT1 (PRMT1^indΔIEC^) did not have any significant effect on the body weight of the animals compared to PRMT1^fl/fl^ mice during the treatment (data not shown). Thus, PRMT1 is critical for EEC homeostasis in the small intestine but not colon of adult mice, although its role, if any, in EECs development during embryogenesis remains to be determined.

### PRMT1 likely regulates EEC number by affecting Neurogenin 3-positive EEC progenitor cells

**Fig. 6:**
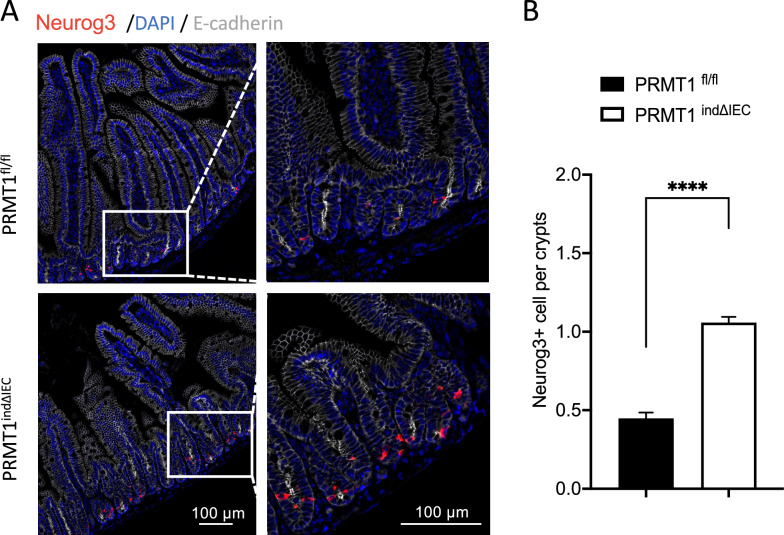
Neurogenin 3-positive progenitor cells are increased in PRMT1-deficient adult small intestine. **A** Neurogenin 3 situ hybridization to detect enteroendocrine progenitor cells in small intestinal sections from PRMT1^indΔIEC^ and PRMT1^fl/fl^ littermates at day 14 after the first tamoxifen injection as diagramed in Fig. 1A. Right panels showed enlarged photo of the boxed area in the left panels. The Neurogenin 3-positive cells were stained red, DNA was stained blue with DAPI, and plasma membrane was stained white with an E-cadherin antibody. Note that Neurogenin3+ cells were located exclusively in the crypts and were much more abundant in PRMT1^indΔIEC^ crypts. **B** Quantification of Neurogenin3+ cells showed that PRMT1 deletion significantly increased enteroendocrine progenitor cells. Multiple sections per animal were analyzed for each group. The values were presented as mean ± SEM with n = 3–4 mice per group. ****p < 0.0001. Scale bars: 100 μm

EECs are derived from neurogenin3 (Neurog3)-positive cells in the small intestine [18, 19, 24, 45]. Given the increased EECs, we observed, not surprisingly, that Neurog3 expression was also upregulated in the small intestinal epithelium of PRMT1^indΔIEC^ mice based on both RNA-seq and RT-qPCR analyses (Fig. 4A, C). In addition, the expression levels of several known downstream target genes of Neurog3, including Neurod1 [20], Pax4/6 [21, 22] Insm1 [23], were also upregulated in the small intestinal epithelium of PRMT1^indΔIEC^ mice (Fig. 4A, C). Thus, PRMT1 knockout might have upregulated Neurog3, leading to increased Neurog3-positive progenitor cells. To test this, we carried out in situ hybridization analysis of Neurog3 expression by using RNA scope [46] on small intestinal sections of mice at day 14 after initiating tamoxifen treatment. In PRMT1^fl/fl^ mice, Neurog3 + cells were rare and expectedly located in the crypts of adult small intestine, where the transit-amplifying (TA) proliferating cells located (Fig. 6A). In contrast, many more Neurog3+ progenitor cells were observed in the PRMT1^indΔIEC^ crypts (Fig. 6A). Quantitative analysis revealed that the Neurog3+ progenitor cells in the PRMT1^indΔIEC^ mice were about 2.4-fold of those in PRMT1^fl/fl^ mice at day 14 after the initiation of tamoxifen treatment (Fig. 6B), similar to the change observed for mature EECs (Fig. 5B). Thus, PRMT1 likely regulates EEC cell number by controlling Neurog3 gene expression and the number of Neurog3+ progenitor cells.

## Discussion

Cell fate specification and differentiation in adult intestine rely on a complex regulatory network of transcription factors and distinct epigenetic landscapes. Whereas many transcription factors involved in enteroendocrine cell differentiation have been identified [17–19, 26, 27, 29, 30], much less is known about epigenetic contributions. Here, we have revealed a novel role for PRMT1, a predominant histone arginine methyltransferase, in enteroendocrine cell fate determination in adult mouse small intestine.

We previously reported that intestinal epithelial PRMT1 knockout altered the structure and epithelial homeostasis in the intestine [38, 39]. Surprisingly, PRMT1 deletion increases cell proliferation in adult intestine, contrary to our expectation based on studies during intestinal remodeling during *Xenopus* metamorphosis, which is equivalent to the period of 3–4 weeks around birth in mouse [40, 47]. This prompted us to investigate the potential molecular mechanism underlying PRMT1 function in adult mouse small intestinal homeostasis. Our RNA-Seq data revealed that tamoxifen-induced PRMT1 knockout in adult mouse intestinal epithelium led to markedly altered transcriptome at day 7 and day 14. Interestingly, there were many transient DEGs at day 7 after initiating tamoxifen treatment, which might be due to the fact that tamoxifen-induced activation of Cre can lead to genome toxicity [41].

Our analysis of the upregulates DEGs in PRMT1^indΔIEC^ crypts at day 14 after the first tamoxifen injection unexpectedly revealed that the most significantly enriched GO terms and KEGG pathways were mostly associated with EECs and that these expression profile in PRMT1 knockout crypts resemble the enrichments due to alteration in the levels of Neurog3, a critical regulator of EEC fate [26]. Consistently, we found that EEC number was increased in the PRMT1 knockout intestine, accompanied by increased expression of enteroendocrine-specific hormones and transcription factors. Importantly, both Neurog3 expression and Neurog3-positive enteroendocrine progenitor cells were also increased in small intestinal crypt of PRMT1 knockout mice, consistent with the report that high Neurog3 gene dosage enforces the commitment of secretory progenitors to an EE lineage [26]. High Neurog3 gene dosage was also reported to constrain goblet cell lineage potential [26, 48]. However, our RNA-seq data did not find any goblet cell markers such as Muc2, Tff3, or goblet cell lineage transcription factors Gfi1 and Spdef [49, 50] among the DEGs (Tables S4), in agreement with our previously studies on Muc2 expression and the total number of goblet cells remain unchanged in PRMT1^indΔIEC^ small intestine [38]. Of note, intestinal epithelium-specific knockout of Neurog3 in mice depletes EECs without affecting nonendocrine epithelial cell types [24, 45], indicating that a complex network of factors controls the secretory progenitor’s fate choice. Our results thus suggest that PRMT1 not only control EEC development via Neurog3 pathway and also likely also affect other intestinal cells via other pathways. Interestingly, our previously study reported a region specific effects of PRMT1 knockout on goblet cells [38]. Here, we observed that PRMT1 also has a region-specific role on EEC differentiation and/or maintenance. Loss of PRMT1 in adult intestinal epithelium increases EECs in the small intestinal but no colon. The small intestine and colon differ significantly, such as the hormones produced, the nutrient absorbed, and the epithelium structure with colon having crypts but lacking villi [1]. The question how these regional differences are established remains to be addressed.

**Fig. 7:**
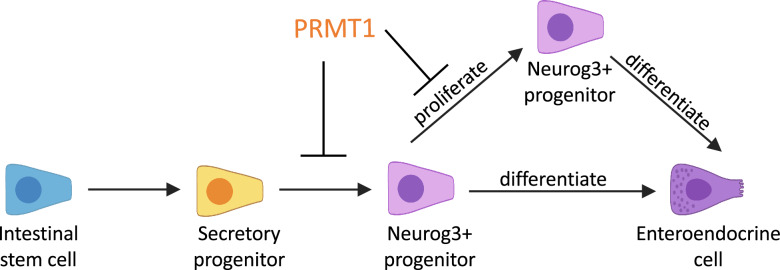
A proposed model for the role of PRMT1 in small intestinal enteroendocrine cell development. PRMT1 suppresses enteroendocrine lineage development, most likely via inhibition of Neurog3 expression through yet unknown mechanism. This may in turn inhibit the formation of Neurog3-postive progenitor cells and/or their proliferation, leading to fewer progenitor cells to differentiate into EECs. Neurogenin 3: Neurog3. EEC: Enteroendocrine cells

Neurog3 has been shown to be expressed transiently in endocrine progenitor cells in the pancreas and controls pancreatic islet cell development and differentiation [51–53]. Interestingly, pancreas-specific PRMT1 knockout mouse embryos exhibit prolonged Neurog3 expression, accompanied by pancreatic hypoplasia after birth [54] and PRMT1 methylation of arginine 65 of Neurog3 has been reported to be required for pancreatic endocrine development of human embryonic stem cell [55]. The loss of PRMT1 leads to Neurog3 accumulation in the cytoplasm and decreases the transcriptional activity of Neurog3 without affecting Neurog3 mRNA level [54, 55]. This seems to be different from our findings in the intestine where the loss of PRMT1 in intestinal epithelium increases Neurog3 mRNA level and Neurog3+ cells, as well as their subsequent differentiation into mature EECs. Thus, PRMT1 appears to regulate Neurog3 to affect EEC development in an organ-dependent manner. In the intestine, PRMT1 suppresses enteroendocrine lineage development. PRMT1 likely inhibits the expression of Neurog3 directly or indirectly. This in turn prevents the formation of Neurog3-postive progenitor cells and/or their proliferation, leading to fewer progenitor cells to differentiate into EECs (Fig. 7). Further studies are needed to determine how PRMT1 affect Neurog3 expression and EEC differentiation in the small intestine.

## Conclusions

Our RNA-Seq analyses have revealed that the epigenetic enzyme PRMT1 plays an important role in regulating the transcriptome of adult mouse small intestine. More importantly, we have uncovered a novel role of PRMT1 in controlling intestinal enteroendocrine cell development and/or homeostasis in adult mice, most likely via inhibition of Neurogenin 3-mediated commitment to EEC lineage. Given the critical roles of gut hormones and peptides in regulating physiological functions, such as nutrient intake, lipid adsorption, and glucose homeostasis, and the involvement of EECs in obesity and diabetes, our results suggest that targeting PRMT1 may offer a novel strategy to control EEC specification to prevent and/or treat human metabolic diseases.

## Materials and methods

### Animals

Mice carrying a LoxP-flanked PRMT1 allele (PRMT1^fl/fl^) and intestinal epithelium-specific PRMT1 knockout mice were generated as described previously [38, 39]. Briefly, PRMT1 knockout mice were generated by crossing PRMT1^fl/fl^ mice with transgenic mice carrying either a constitutively active Cre recombinase under the control of the villin promoter (Vil-Cre) (The Jackson Lab), or tamoxifen-inducible Cre, active CreER^T2^, under the same promoter (Vil-CreERT2) (The Jackson Lab). To active CreER^T2^, adult PRMT1^fl/fl^ mice and mice of interest (PRMT1^fl/fl^; vil-Cre^ERT2^, henceforth referred to as PRMT1^indΔIEC^) were injected intra-peritoneally (IP) with tamoxifen (T5648, Sigma–Aldrich; 2 mg each mouse) for up to 5 consecutive days. The mice were euthanized at the indicated time points after the first tamoxifen injection at day 0 (Fig. 1A). All mice used in this study were age- and gender- matched between PRMT1 knockout and control. All animal care and procedures were approved by the Animal Use and Care Committee of Eunice Kennedy Shriver National Institute of Child Health and Human Development (NICHD), National Institutes of Health (NIH).

### Genotyping

Mouse tail tips were used to isolate genomic DNA with QuickExtract DNA extraction solution (25887, Biosearch technologies). The DNA was used for PCR genotyping with primers for PRMT1, Cre and CreERT2 as described previously [38, 39]. The PCR products were analyzed with 2% agarose gel electrophoresis to determine the genotype based on the sizes of the PCR products.

### Histological processing and immunofluorescent staining

Isolated intestine was flushed with ice-cold 1X phosphate-buffered saline (PBS) and fixed in 10% neutral buffered formalin (R04586, Sigma–Aldrich) at room temperature overnight, then transferred into 70% ethanol, processed with a tissue processor (Excelsior AS Tissue Processor; Thermo Fisher Scientific), followed by embedding in paraffin and then cutting into 5 µm sections.

Chromogranin A (ab15160, Abcam; 1:200 dilution) and serotonin (ab66047, Abcam, 1:100 dilution) immunofluorescence analyses were performed on paraffin-embedded sections (5 m) as described previously [38]. The fluorescent pictures for different colors and different sections were taken by using a microscope under the same settings and then analyzed with ImageJ software.

### RNA scope in situ hybridization

RNA scope, high-resolution RNA in situ hybridization [46], was performed on 5 µm formalin-fixed, paraffin-embedded sections by using RNAscope Multiplex Fluorescent Reagent Kit (323100, ACDBio). The RNAscope probes used were Neurog3 (422401, ACDBio), and the positive control probe Ppib (313911, ACDBio), and the negative control probe DapB (310043, ACDBio). Alexa Fluor® 488 Mouse anti-E-Cadherin (560061, BD Bioscience) was used for visualizing plasma membrane.

### Isolation of mouse intestinal crypts

Intestinal crypts were isolated as previous described [38] from small intestine and colon of 8–12 weeks old PRMT1^fl/fl^ and PRMT1^indΔIEC^ mice at different time point after tamoxifen injection (Fig. 1A). Briefly, mouse small intestine and colon were isolated, opened longitudinally, and then cut into small pieces. The small pieces were washed twice with cold PBS and then incubated with 20 mM EDTA in PBS on ice for 40 min. After removal of EDTA, the tissue pieces were vigorously suspended by using a 10-ml pipette with cold PBS containing 0.1% BSA. The supernatant, which was enriched with crypts, was filtered through 70 μm cell strainer (352350, Corning) and centrifuged at 300*g* for 3 min. The pellets were resuspended in TRIzol™ Reagent (15596026, Invitrogen) for RNA isolation.

### RNA-Seq and data analysis

Three biological replicates from the PRMT1^fl/fl^ and PRMT1^indΔIEC mice^ group for each time point after tamoxifen injection (Fig. 1A) were used for RNA extraction. Total RNA from small intestinal crypts were isolated as described above and extracted by using Direct-zol™ RNA Miniprep (R2052, ZYMO Research) according to the manufacturer’s instructions. The RNA samples were then sent to the NICHD Molecular Genomics Core for library preparation and sequencing. Libraries was prepared by using the Illumina TruSeq total RNA prep kit with Ribo-zero kit at the step of ribodepletion and sequenced on the Illumina NovaSeq 6000 platform to obtain 100 bp paired-end reads for each of the 3 biological replicates, respectively. For each sample, reads were identified and mapped onto the mm10 (Gencode M26) assembly of mouse genome by using STAR software (v2.7.3). Quantification of gene expression was determined by HTSeq software featurecounts v1.6.4 and gene annotations from Gencode release M26. Normalization of read counts and differential gene expression analysis between PRMT1^fl/fl^ and PRMT1^indΔIEC^ samples were further performed by using the R package DESeq2 (v1.38). Genes with an adjusted p-value (p-adj) < 0.05 were considered as significantly differentially expressed accepting a 5% FDR. Principal component analysis (PCA) was performed by using the R package DESeq2 (v1.38) package. DEGs between PRMT1 knockout and PRMT1^fl/fl^ mice were plotted on a MA plot by using the DESeq2 ggpubr package. To identify enriched biological processes and pathways among the DEGs, the GO and KEGG analyses were performed with the bioconductor ClusterProfiler package (v4.6.0). Venny 2.1 (https://bioinfogp.cnb.csic.es/tools/venny/) was used for visualizing the overlapped genes Venn diagrams. The raw datasets were deposited in Gene Expression Omnibus (GEO) repository (GSE263245).

### RT-qPCR analysis

One μg total RNA was reverse-transcribed into cDNA by using High-Capacity cDNA Reverse-Transcription Kit (4368814, Applied Biosystems). The qPCR was then performed by using SYBR Green PCR Master mix (A25742, Applied Biosystems) in a total volume of 10 μl on Step One Plus Real-Time PCR System (Applied Biosystems) with indicated primers (Table S9). Fold changes were calculated by using the ΔΔCT method with $\beta$-actin used as a control.

### Statistical analysis

Statistical significance for the differences between samples was determined by using a two-tailed unpaired Student's t-test. Except for RNA-Seq, all experiments were repeated for at least two times. For the analysis of intestinal cross-sections, individual cross-sections instead of individual animals were used as samples for the Student’s t-test. Data were presented as the mean ± SEM (the standard error of the mean). Prism 10 from GraphPad software was used to calculate P values and plot figures. Differences with P values of less than 0.05 were considered significant: *p < 0.05, **p < 0.01, ***p < 0.001, ****p < 0.0001, ns: no significant.

### Supplementary Information

Supplementary Material 1.

Supplementary Material 2.

## Data Availability

The raw datasets were deposited in Gene Expression Omnibus (GEO) repository (GSE263245).

## Abbreviations

PRMT1: Protein arginine methyltransferase 1
ISCs: Intestinal stem cells
EECs: Enteroendocrine cells
Neurog3: Neurogenin 3
RNA-Seq: RNA-Sequencing
DEGs: Differentially expressed genes
GO: Gene Ontology
KEGG: Kyoto Encyclopedia of Genes and Genomes
RT-qPCR: Quantitative reverse transcription polymerase chain reaction
CHGA: Chromogranin A
