# Supplementary material for: Protein arginine methyltransferase 1 regulates mouse enteroendocrine cell development and homeostasis
Source: Cell Biosci. 2024 Jun 4;14:70. doi: 10.1186/s13578-024-01257-x (PMC11151601; doi:10.1186/s13578-024-01257-x)
Supplement: Supplementary file 1 — Supplementary Material 1. [file 13578_2024_1257_MOESM1_ESM.pdf]

Fig. S1.

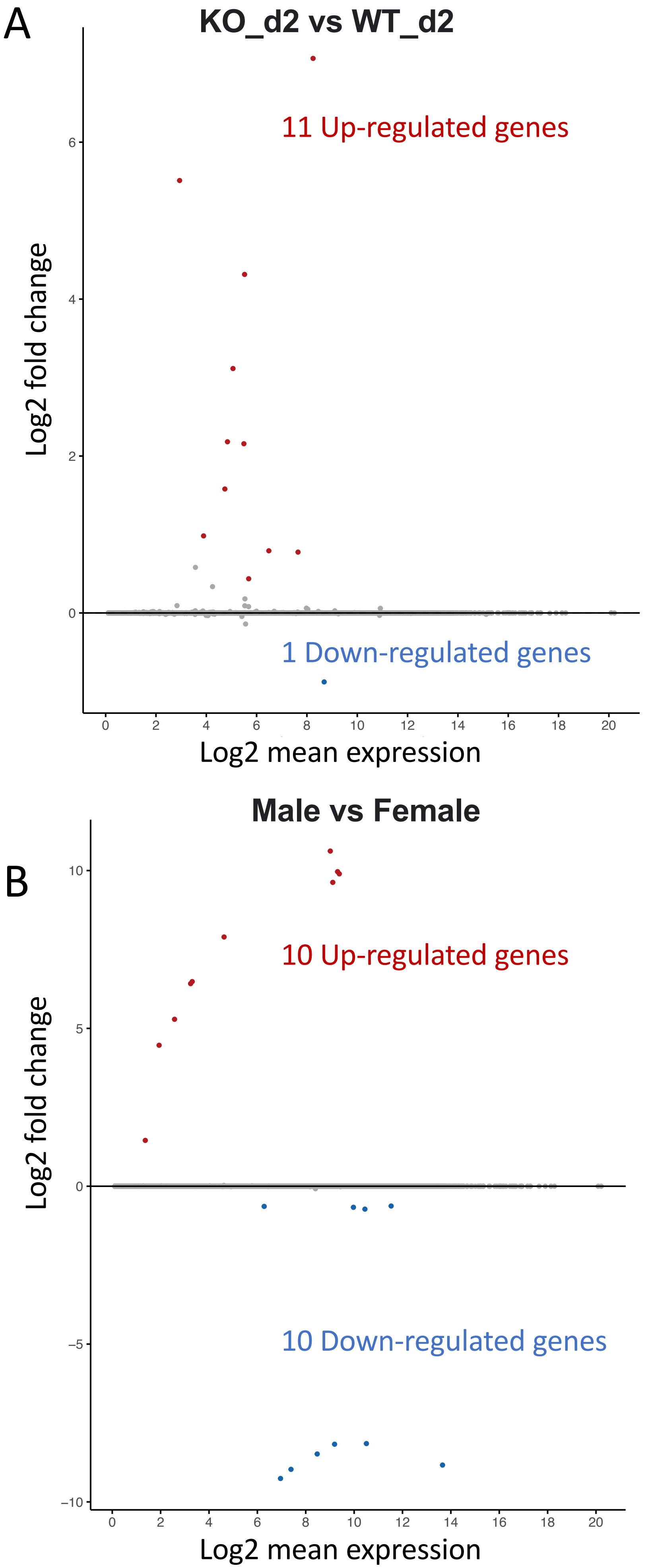

Additional file 1: Fig. S1. Gene expression profiling of the small intestinal crypts of adult wild type (WT) or induced PRMT1 knockout (PRMT1$^{ind\Delta IEC}$) mice. RNA-Seq analyses were carried out on the small intestinal crypts of wild type or adult PRMT1$^{ind\Delta IEC}$ mice 2 days after the initiation of tamoxifen treatment. **(A)** There are few differentially expressed genes (DEGs) between wild type (WT_d2) and PRMT1$^{ind\Delta IEC}$ (KO_d2) 2 days after initiating KO with tamoxifen treatment. MA plot visualizing the log2-fold change (M values) between wild type and KO expression based on log2-mean expression levels (A values). Red and blue dots represent significantly (Adjusted p-value <0.05) up- and down-regulated genes, respectively, in PRMT1$^{ind\Delta IEC}$. **(B)** There are a number of DEGs between male and female mouse intestine. Red and blue dots represent significantly (Adjusted p-value <0.05) up- and down-regulated genes, respectively, in the male intestine compared to female intestine.

Fig. S2.

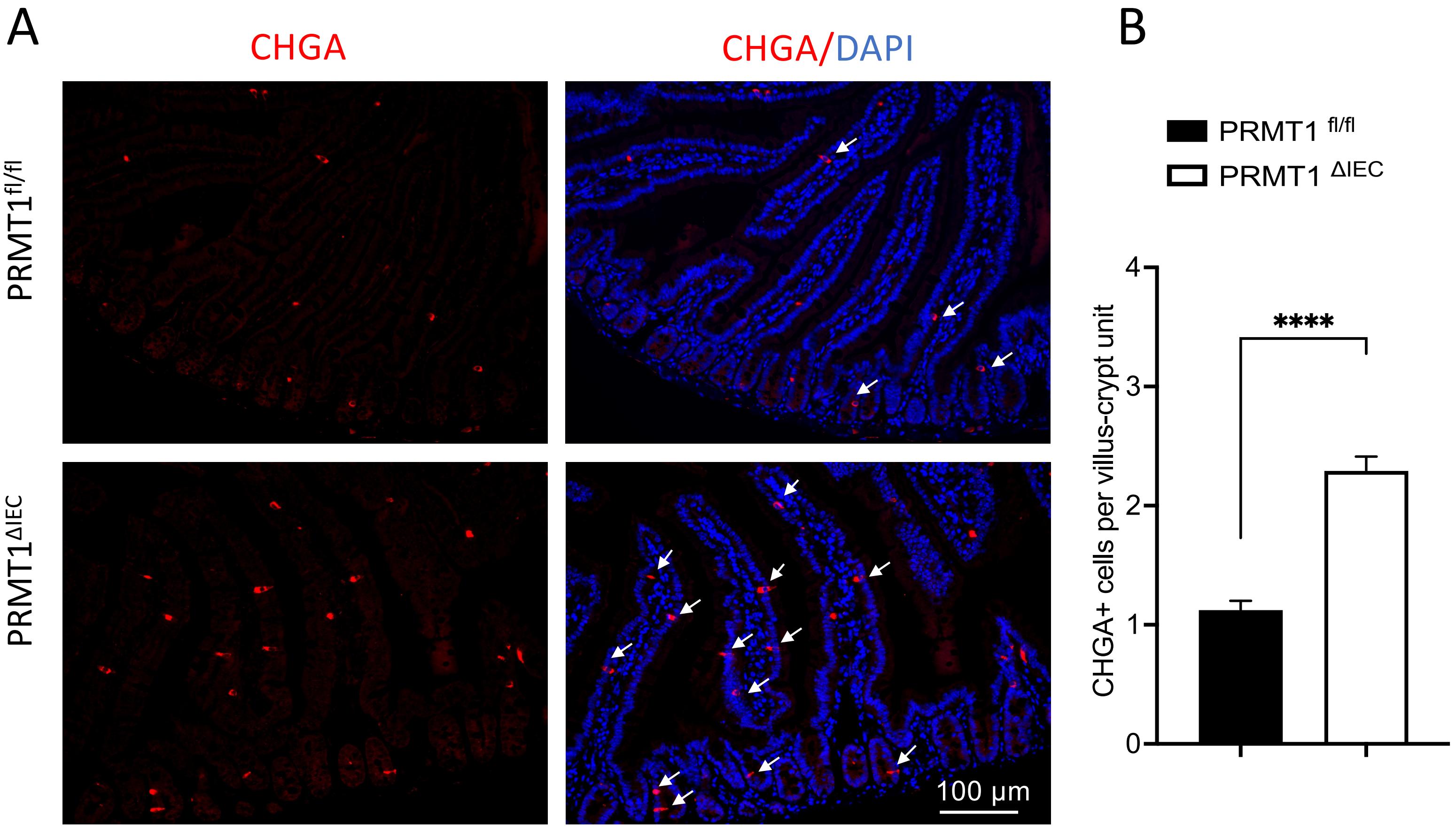

Additional file 2: Fig. S2. Intestinal epithelium-specific knockout of PRMT1 (PRMT1$^{\Delta IEC}$) increased enteroendocrine cells (EEC) in adult small intestine. (A) Immunofluorescent staining for CHGA, an EEC marker, in small intestinal cross-sections of adult PRMT1$^{\Delta IEC}$ and PRMT1$^{fl/fl}$ littermates. The CHGA-labeling stained EEC cells red (arrows), and the DNA was stained blue with DAPI. Note that PRMT1$^{\Delta IEC}$ mice carrying a constitutively active Cre recombinase under the control of villin promoter (Vil-Cre), expressing Cre in the intestinal epithelial cells during embryonic development and throughout adulthood. **(B)** Quantification of CHGA+ cells showed that PRMT1 deletion dramatically increased EEC numbers in adult intestine. Multiple sections per animal were analyzed for each group. The values were presented as mean ± SEM with n=3-4 mice per group. ****$p < 0.0001$. Scale bars indicate 100 µm.

Fig. S3.

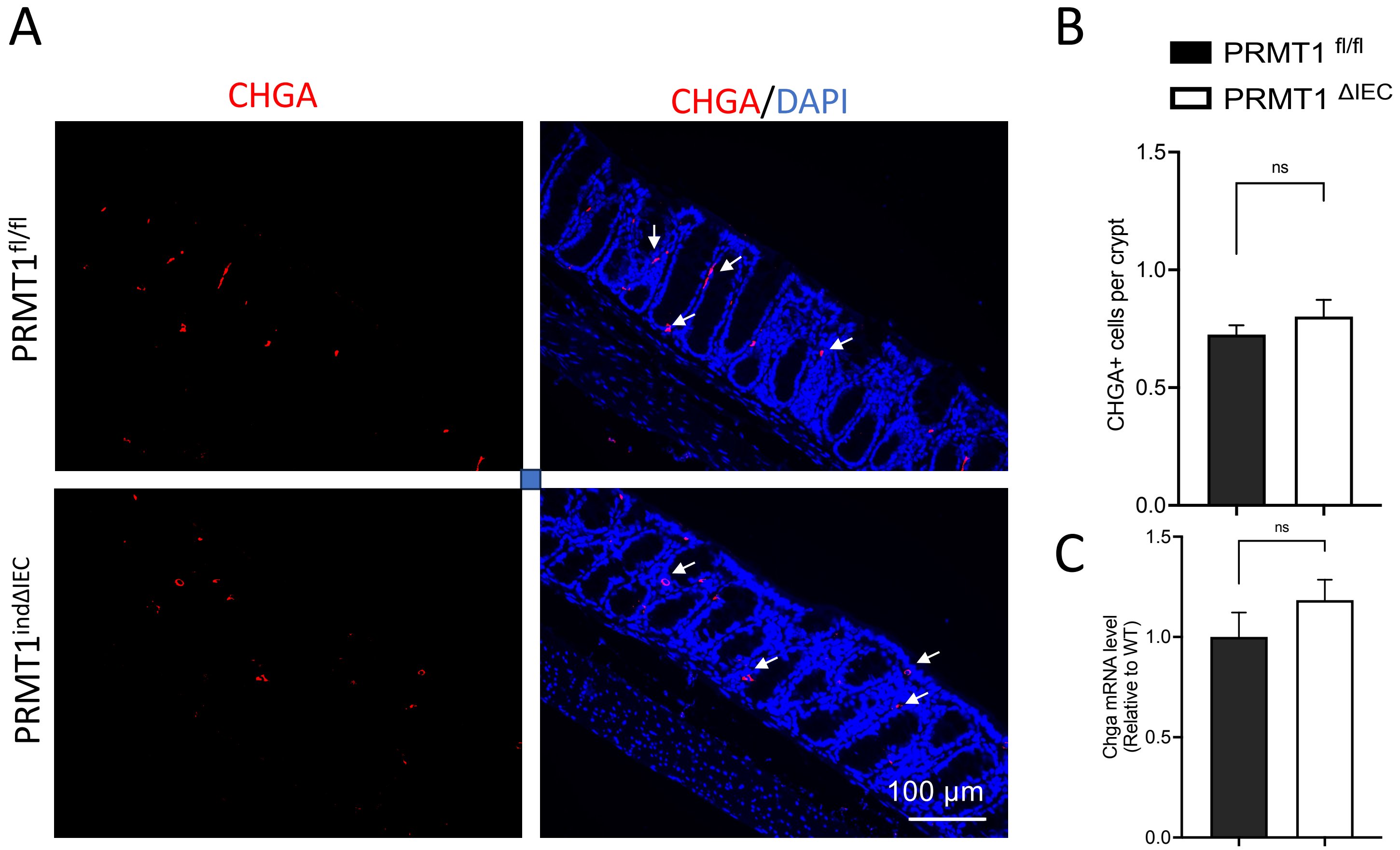

Additional file 3: Fig. S3. Intestinal epithelium-specific deletion of PRMT1 does not affect enteroendocrine cell (EEC) number in the adult colon. (A) Immunofluorescent staining for CHGA, an EEC marker, in colonic sections from $PRMT1^{ind\Delta IEC}$ and $PRMT1^{fl/fl}$ littermates at day 14 after the first tamoxifen injection. The CHGA-labeling stained EEC cells red (arrows), and the DNA was stained blue with DAPI. **(B)** Quantification of CHGA+ cells showed that PRMT1 deletion did not significantly change EEC numbers in the adult colon. Multiple sections per animal were analyzed for each group. Scale bars indicate 100 μm. **(C)** Analyses of Chga expression in the colonic epithelial cells of $PRMT1^{ind\Delta IEC}$ and $PRMT1^{fl/fl}$ mice by RT-qPCR. The values were presented as mean ± SEM with n=3-4 mice per group. ns: no significant.
